# Identification and Potential Regulatory Properties of Evolutionary Conserved Regions (ECRs) at the Schizophrenia-Associated MIR137 Locus

**DOI:** 10.1007/s12031-016-0812-x

**Published:** 2016-08-15

**Authors:** Olympia Gianfrancesco, Daniel Griffiths, Paul Myers, David A. Collier, Vivien J. Bubb, John P. Quinn

**Affiliations:** 1 Department of Molecular and Clinical Pharmacology, Institute of Translational Medicine, The University of Liverpool, L69 3BX, Liverpool, UK; 2 Eli Lilly and Company Limited, Windlesham, Surrey UK

**Keywords:** Schizophrenia, microRNA-137, Gene regulation, Gene expression, Transcription, Genetics

## Abstract

Genome-wide association studies (GWAS) have identified a region at chromosome 1p21.3, containing the microRNA MIR137, to be among the most significant associations for schizophrenia. However, the mechanism by which genetic variation at this locus increases risk of schizophrenia is unknown. Identifying key regulatory regions around MIR137 is crucial to understanding the potential role of this gene in the aetiology of psychiatric disorders. Through alignment of vertebrate genomes, we identified seven non-coding regions at the MIR137 locus with conservation comparable to exons (>70 %). Bioinformatic analysis using the Psychiatric Genomics Consortium GWAS dataset for schizophrenia showed five of the ECRs to have genome-wide significant SNPs in or adjacent to their sequence. Analysis of available datasets on chromatin marks and histone modification data showed that three of the ECRs were predicted to be functional in the human brain, and three in development. In vitro analysis of ECR activity using reporter gene assays showed that all seven of the selected ECRs displayed transcriptional regulatory activity in the SH-SY5Y neuroblastoma cell line. This data suggests a regulatory role in the developing and adult brain for these highly conserved regions at the MIR137 schizophrenia-associated locus and further that these domains could act individually or synergistically to regulate levels of MIR137 expression.

## Introduction

Meta-analyses of schizophrenia genome-wide association studies (GWAS) data have identified a locus on chromosome 1p21.3 (chr1:98,298,371–98,581,337, GRCh37/hg19) to be among the most significantly associated regions for schizophrenia (Ripke et al. 2013; Schizophrenia Psychiatric Genome-Wide Association Study 2011). The microRNA MIR137 is present within this locus, and subsequent work has revealed transcripts from other schizophrenia-associated loci as targets of MIR137 (Collins et al. 2014; Kim et al. 2012; Kwon et al. 2013), thereby suggesting MIR137-mediated regulation of a larger network of genes and pathways relevant to mental illness. MIR137 is highly expressed in the brain and is known to function in neural development and adult neurogenesis (Smrt et al. 2010; Szulwach et al. 2010), as well as regulating synaptic plasticity (Siegert et al. 2015).

Sequencing of the MIR137 locus by Duan et al. revealed 133 new variants which are enriched in non-coding sequences, and one of these, a rare enhancer SNP 1:g.98515539 A> T, was associated with schizophrenia and reduced enhancer activity of its flanking sequence, predicting lower expression of MIR137 (Duan et al. 2014). The authors predicted the location of these SNPs as potential transcriptional regulators from ENCODE data, including the use of histone marks such as H3K4me1. We have previously shown that comparative genomics overlaid on SNP association data can identify non-coding DNA which may be involved mechanistically in conditions such as depression, alcoholism and obesity (Davidson et al. 2011; Davidson et al. 2015; Hing et al. 2012). Using a similar strategy to better understand the genomic regulatory architecture around the MIR137 locus, we used the evolutionary conserved region (ECR) and UCSC Genome Browsers to align and compare multiple vertebrate genomes to identify and prioritise conserved domains of interest. These regions were further analysed using publically available schizophrenia GWAS and epigenetic data, from the Psychiatric Genomics Consortium and the Roadmap Epigenomics Consortium, respectively. We herein identify seven functional ECRs at the schizophrenia-associated MIR137 locus and characterise their activities in vivo through analysis of epigenetic data, and in vitro by dual luciferase reporter assays.

## Materials and Methods

### Bioinformatic Analysis

Bioinformatic analysis was carried out using ECR Browser (http://ecrbrowser.dcode.org) and UCSC Genome Browser (http://genome.ucsc.edu) to identify ECRs of interest at the MIR137 locus. UCSC genome browser was also used to overlay Psychiatric Genomics Consortium’s SCZ2 schizophrenia GWAS data (https://www.med.unc.edu/pgc/downloads) and access GenBank human EST data. Additionally, the Broad Institute’s Ricopili tool was used to visualise schizophrenia GWAS SNPs from the ‘PGC_SCZ52_may13’ dataset across the MIR137 locus (http://www.broadinstitute.org/mpg/ricopili/).

LD analysis used SNP genotype data from the CEU/CEPH cohort, spanning the region chr1:98,498,912–98,595,043 (GRCh37/hg19) and downloaded from the HapMap Genome Browser,

(http://hapmap.ncbi.nlm.nih.gov/), release #28. LD analysis was performed using Haploview 4.2,

(www.broad.mit.edu/mpg/haploview/) with the following parameters: Hardy-Weinberg *p*-value cut-off, 0.001; minimum genotype cut-off, 75 %; maximum number of Mendel errors, 1; minimum minor allele frequency, 0.01) and pair-wise tagging analysis performed (*r*^2^ threshold, 0.8). Haplotype blocks were determined using 95 % confidence intervals (Gabriel et al. 2002). HaploReg V4.1 (http://www.broadinstitute.org/mammals/haploreg/haploreg.php) (Ward and Kellis 2012) was used to access chromatin state and histone data from the Roadmap Epigenomics Consortium (Roadmap Epigenomics et al. 2015) in order to assess potential activity of ECRs in vivo.

### Generation of pGL3P Reporter Gene Constructs

All evolutionary conserved regions in this study were amplified by PCR from pooled mixed-gender human genomic DNA preparations (Promega) using Phusion High-Fidelity DNA Polymerase (New England Biolabs). Amplified fragments were cloned into the pGL3P luciferase reporter vector (Promega) using Gibson Assembly Cloning Kit (New England Biolabs) as described in manufacturer’s protocol, and transformed into XL10-Gold ultracompetent cells (Agilent Technologies). In order to allow directional cloning, primers used for amplification included tails of 16–20 bp, complementary to the sequence flanking the cut site of the vector into which the fragments were to be cloned.

The following primer sets were used (5′ ➔ 3′). Underlined sequence indicates 16–20-bp flanking region complementary to cloning vector:

MIR 1 Fwd*:* GCAGTGGCTGTAAGATGAGGA

MIR 1 Rev*:* AGAGGCCTGGAGTCTGTGAC

MIR 2 Fwd*:* CCCCATGATGTTCTCATACCA

MIR 2 Rev.: TACAGCCACTGCAAATACGG

MIR 3 Fwd*:*AGCTCTTACGCGTGCTAGCTGCACTTTGCATTCCTC.

MIR 3 Rev*:*AGATCGCAGATCTCGAGCTCACACTTCCTAACTGGT

MIR 4 Fwd*:*AGCTCTTACGCGTGCTAGTGCCCTTGTCTAATGAA

MIR 4 Rev*:*AGATCGCAGATCTCGAGCATTCAGGACTCTAGTCT

MIR 5 Fwd*:*AGCTCTTACGCGTGCTAGAGAAGAGGATTTGTGGGCTAC

MIR 5 Rev*:*AGATCGCAGATCTCGAGGGCTTGGGATACCTGACAATTAGCAAC

MIR 6 Fwd: AGCTCTTACGCGTGCTAGAGCCTCTACAATTCAGGA

MIR 6 Rev: AGATCGCAGATCTCGAGCCAAGGACACTGAGGATAT

MIR 7Fwd*:*CGAGCTCTTACGCGTGCTAGACATTCTTGATTTGCATAA

MIR 7Rev: AGATCGCAGATCTCGAGTGCTTCAGTGTAACTACTG

Genomic co-ordinates (GRCh37/hg19) for the ECR fragments amplified are as follows:

MIR 1: chr1:98499831-98500934 (1104 bp)

MIR 2: chr1:98500923-98502814 (1892 bp)

MIR 3: chr1:98525381-98525895 (515 bp)

MIR 4: chr1:98538705-98540267 (1563 bp)

MIR 5: chr1:98552809-98554083 (1275 bp)

MIR 6:chr1:98567339-98567854 (516 bp)

MIR 7: chr1:98592252-98592661 (410 bp)

Cloning of inserts was verified by bi-directional sequencing using standardised primers.

### Culture of SH-SY5Y Human Neuroblastoma Cells

Human neuroblastoma cell line, SH-SY5Y (ATCC CRL-2266), was grown and maintained in a 50:50 mix of Minimal Essential Medium Eagle (Sigma) and Nutrient Mixture F-12 Ham (Sigma), supplemented with 10 % foetal bovine serum (ThermoScientific), 1 % penicillin/streptomycin (100 U/ml, 100 mg/ml; Sigma), 1 % (*v*/*v*) 200 mM L-glutamine (Sigma), and 1 % (*v/v*) 100 mM sodium pyruvate (Sigma). Cells were incubated at 37 °C with 5 % CO_2_.

### Transfection and Dual Luciferase Assays

SH-SY5Y cells were seeded at approximately 100,000 cells per well in 24-well plates. After overnight incubation, cells were co-transfected with 2 μg reporter DNA and 20 ng pMLuc-2 (a TK renilla luciferase vector used as an internal control for normalisation; Novagen, USA) using TurboFect Transfection Reagent (ThermoScientific/Fermentas), according to manufacturer’s protocol.

Luciferase reporter assays were performed 48 h post-transfection. Luciferase activity of reporter constructs was measured using a Dual Luciferase Reporter Assay System (Promega) using 20 μl lysate from transfected cells according to manufacturer’s instructions. Assays were carried out on a Glomax 96-well microplate Luminometer (Promega). Two-tailed *t* test for significance compared fold change in luciferase expression to the vector containing the minimal promoter alone (*P < 0.1, **P < 0.01, ***P < 0.001) *N* = 4.

## Results

### Identification of ECRs at the MIR137 Locus

**Fig. 1:**
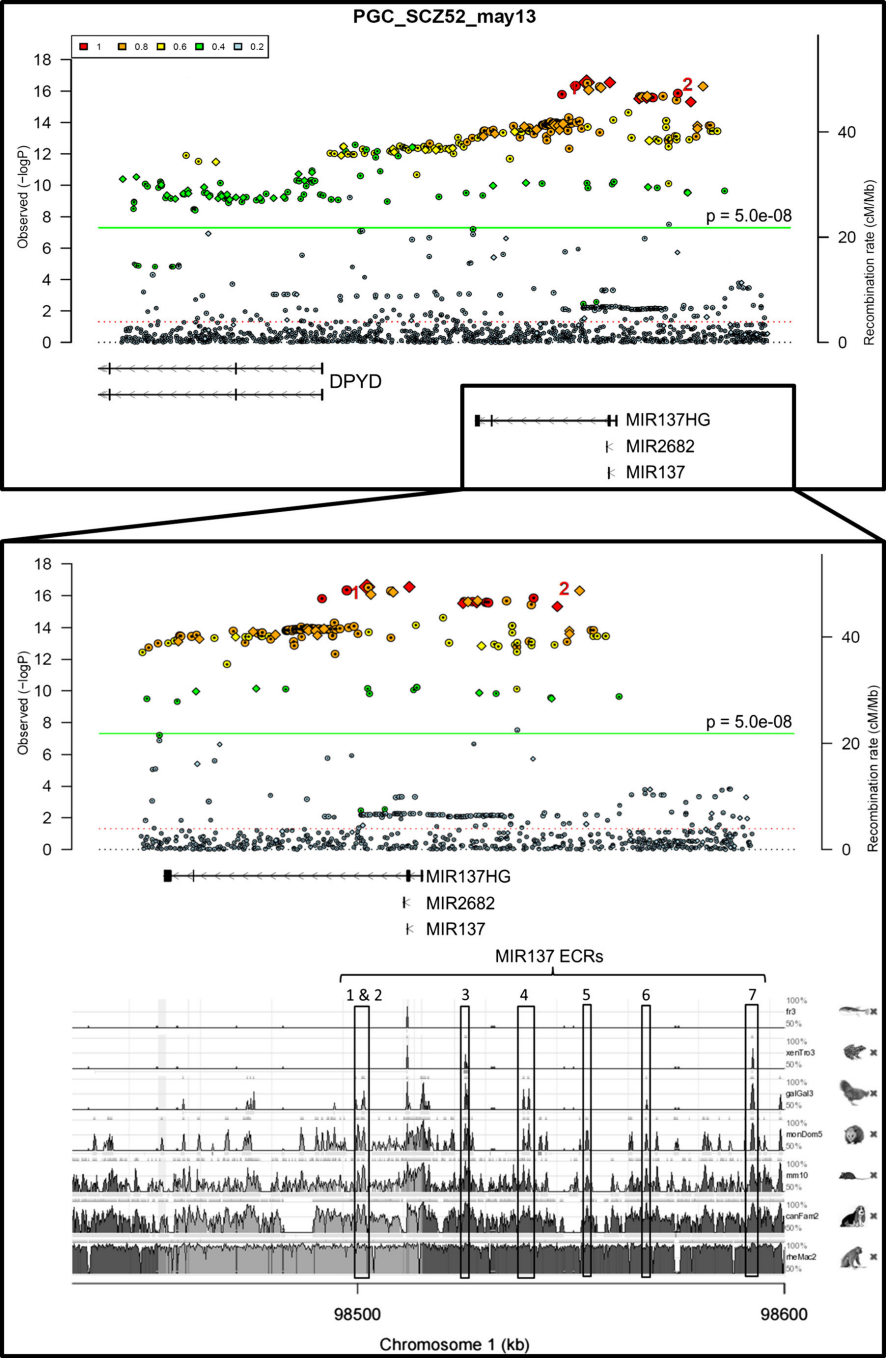
Schizophrenia genome-wide associated SNPs and evolutionary conserved regions (ECRs) at the MIR137 locus. Visualisation of the Psychiatric Genomics Consortium’s May 2013 schizophrenia GWAS data across the full schizophrenia-associated locus (chr1:98298371-98595000, GRCh37/hg19), using the Broad Institute’s Ricopili tool. The boxed region including MIR137 and upstream regions (chr1:98448000-98595000; GRCh37/hg19) displays the highest signal at this locus in terms of association for schizophrenia, suggesting that MIR137 and potential upstream regulatory regions are likely to be the causal association at this locus. The boxed region is expanded below, and schizophrenia GWAS SNP data overlaid onto species conservation data from the ECR browser. Statistically significant schizophrenia GWAS SNPs are represented by *coloured circles* or *diamonds* above the green line (*p* = 5.0e-08). Red numbers 1 and 2 highlight the schizophrenia GWAS SNPs rs1625579 and rs1198588, respectively. ECRs selected for study are boxed and numbered. Conserved peaks between boxes 2 and 3 represent the microRNA and its proximal promoter, which were not included in this analysis, but highlight conservation as an indicator of functionality

Genome-wide significant variants associated with complex disorders are overwhelmingly in non-coding regions of the genome and potentially exert their effect through regulation of the transcriptome, rather than via primary changes in coding sequence. Identifying non-coding evolutionary conserved regions (ECRs) is one method of determining regulatory domains that may influence gene expression at the MIR137 locus. We used the ECR Genome Browser to screen for ECRs across this region based on a seven-way alignment of vertebrate genomes. This identified four highly conserved regions upstream of MIR137 (MIR 3, 4, 6 and 7). Three additional ECRs were selected for study due to their proximity to highly significant schizophrenia GWAS SNPs, namely rs1625579 and rs1198588; two were intronic, MIR 1 and 2, and one was upstream of MIR137, MIR 5. ECRs are herein named MIR 1–7 (Fig. 1).

### Regions Encompassing MIR ECRs 1, 2, 3, 5 and 7 Contain Schizophrenia GWAS SNPs

Following identification of the above ECRs, bioinformatic analysis was carried out using the UCSC Genome Browser to align the Psychiatric Genomics Consortium’s latest schizophrenia GWAS data (PGC2, accessible at: http://www.med.unc.edu/pgc/downloads) over the MIR137 locus ECRs.

**Fig. 2:**
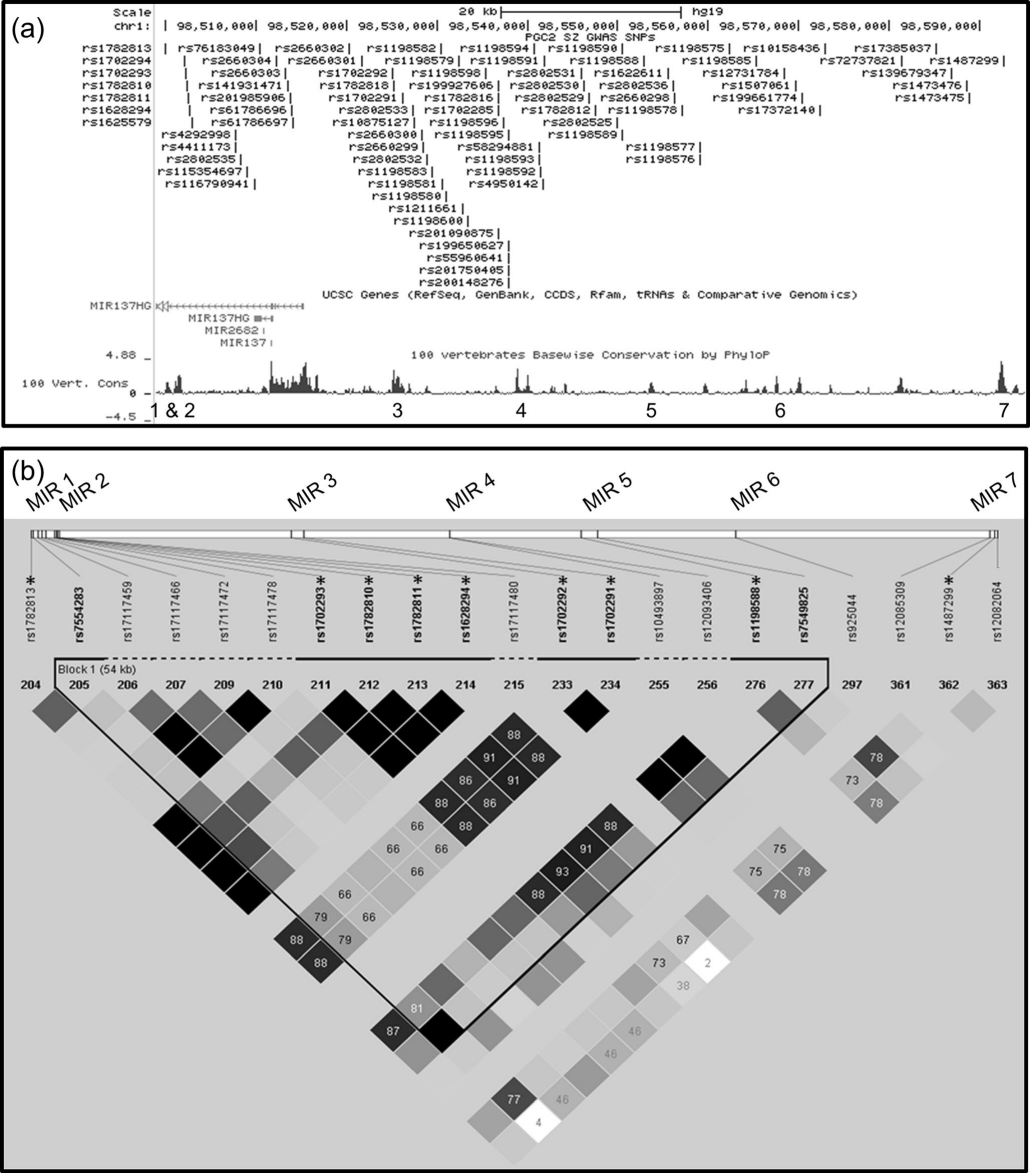
Linkage disequilibrium (LD) analysis of SNPs across the MIR137 ECRs: **a**) Schematic of MIR137 and upstream region containing the ECRs of interest, with PGC2 schizophrenia GWAS data overlaid, showing 80 schizophrenia GWAS SNPs across this locus. ECRs are numbered 1–7 and visualised as peaks on the 100 vertebrates base wise conservation track from the UCSC Genome Browser. **b**) LD analysis of SNPs at the MIR ECRs was carried out using SNP genotype data from the HapMap CEU cohort, with D’ values shown. Data for 9 of the schizophrenia GWAS SNPs (starred*) was available in this cohort, and analysed alongside data for non-GWAS HapMap SNPs within the same ECRs. In this analysis, a haplotype block was found to link SNPs from ECRs MIR 1–5, with schizophrenia GWAS SNPs at MIR 1, 2, 3 and 5 in strong linkage disequilibrium with each other

PGC2 data showed that the strongest GWAS signal at this locus was across MIR137 itself and extended significantly into the upstream region (Fig. 1). Current data shows 80 schizophrenia GWAS SNPs across the 96.1-kb region containing the seven selected ECRs (chr1:98,498,912–98,595,043; GRCh37/hg19), with three of these GWAS SNPs being within MIR 3, one in MIR 7, and an additional seven GWAS SNPs adjacent to MIR ECRs 1, 2 and 5 (Fig. 2).

Linkage disequilibrium analysis was carried out using the nine ECR schizophrenia GWAS SNPs for which data was available in the HapMap CEU cohort, with additional non-GWAS HapMap SNPs from these regions included (Fig. 2). Haplotype blocks were identified in HaploView using the default parameters as specified by Gabriel et al. (Gabriel et al. 2002). Of the GWAS SNPs used in this analysis, seven were found to be in a haplotype block, linking MIR ECRs 1, 2, 3 and 5 over a region of ~54.9 kb (chr1:98,499,795–98,554,659). This analysis also showed that schizophrenia GWAS SNPs within the ECRs at the MIR137 locus are preferentially in linkage disequilibrium with each other, whereas non-GWAS SNPs in the same ECRs are not. This indicates that the schizophrenia-associated SNPs may function in combination and mediate risk through a shared or combined mechanism.

### Bioinformatic Analysis of ECR Function In Vivo

**Fig. 3:**
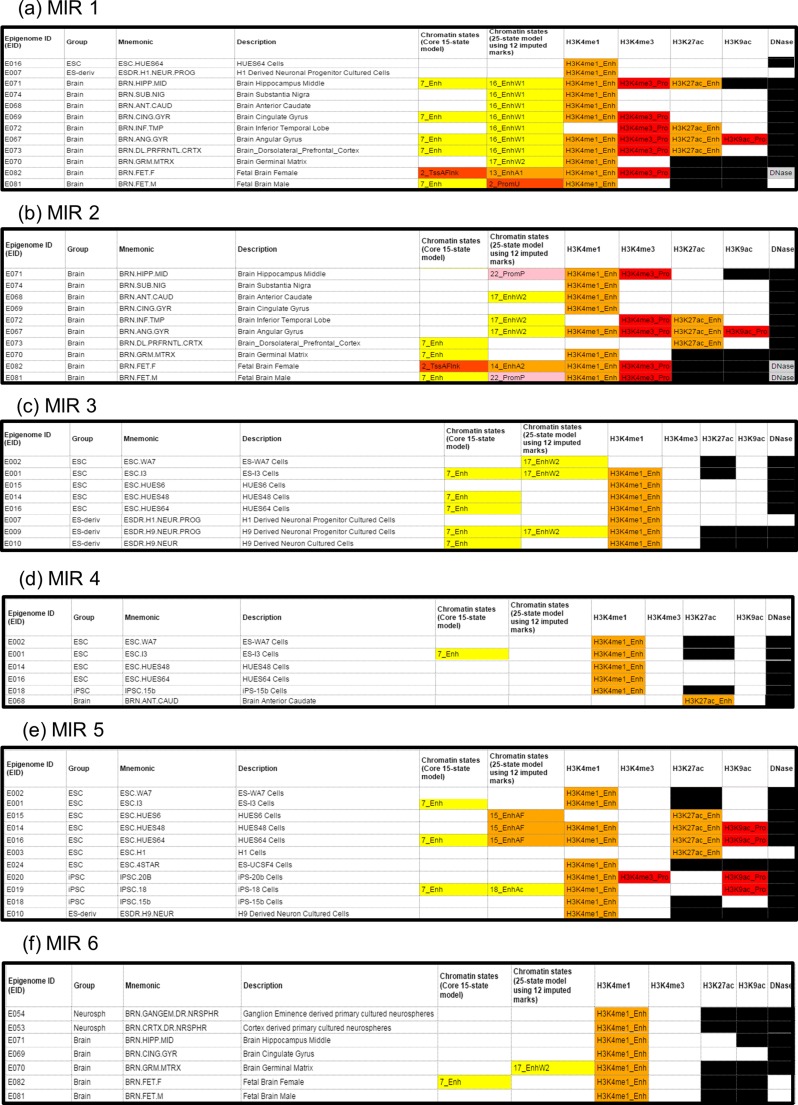
Chromatin state and histone modifications at MIR ECRs in the brain and embryonic tissues. Data from the Roadmap Epigenomics Consortium shows that MIR 1, 2 and 6 (3a, b and f) are active regulators of transcription in many areas of the human brain, whereas MIR 3, 4, and 5 (3c, d and e) are active in embryonic and induced pluripotent stem cells, and may function to regulate transcription at this locus during development. Chromatin states: *Enh* = enhancer, *EnhA1/2* = active enhancer 1 or 2, *EnhAc* = enhancer acetylation only, *EnhAF =* active enhancer flank, *EnhW1/2* = weak enhancer 1 or 2, *PromP* = poised promoter, *PromU* = promoter upstream transcriptional start site, *TssA* = active transcription start site, *TssAFlank* = flanking active transcriptional start site. Histone modifications: *Enh* = enhancer, *Pro =* promoter. *Black* = no available data

HaploReg V4.1 was used to access chromatin structure and histone modification data from the Roadmap Epigenomics Consortium. This data showed that the ECRs MIR 1 and 2 are predicted to be enhancers in the brain, displaying H3K4me1, H3K4me4 and H3K27ac histone modifications across multiple brain regions, which are indicative of active regulation and transcription at these loci. Methylation data for MIR 6 also shows H3K4me1 marks at a number of brain regions including the hippocampus, cingulate gyrus, germinal matrix and across the foetal brain, consistent with this ECR being an active or poised transcriptional regulator in these tissues (Fig. 3a, b, f).

Conversely, analysis of data across MIR 3, 4 and 5 predicted these regions to be active regulators during development, with histone modifications and chromatin state data consistent with transcriptional regulatory activity in multiple embryonic and induced pluripotent stem cell lines, as well as in stem cell-derived neuronal progenitor or cultured neuron cells. In addition to H3K4me1 histone modifications, H3K27ac and H3K9ac marks are also seen across MIR 5 in multiple embryonic and induced pluripotent stem cell lines, suggesting active transcription from a nearby promoter during development (Fig. 3c, d, e). No relevant data was seen over the MIR 7 ECR locus.

### Transcriptional Regulatory Activity of MIR ECRs

**Fig. 4:**
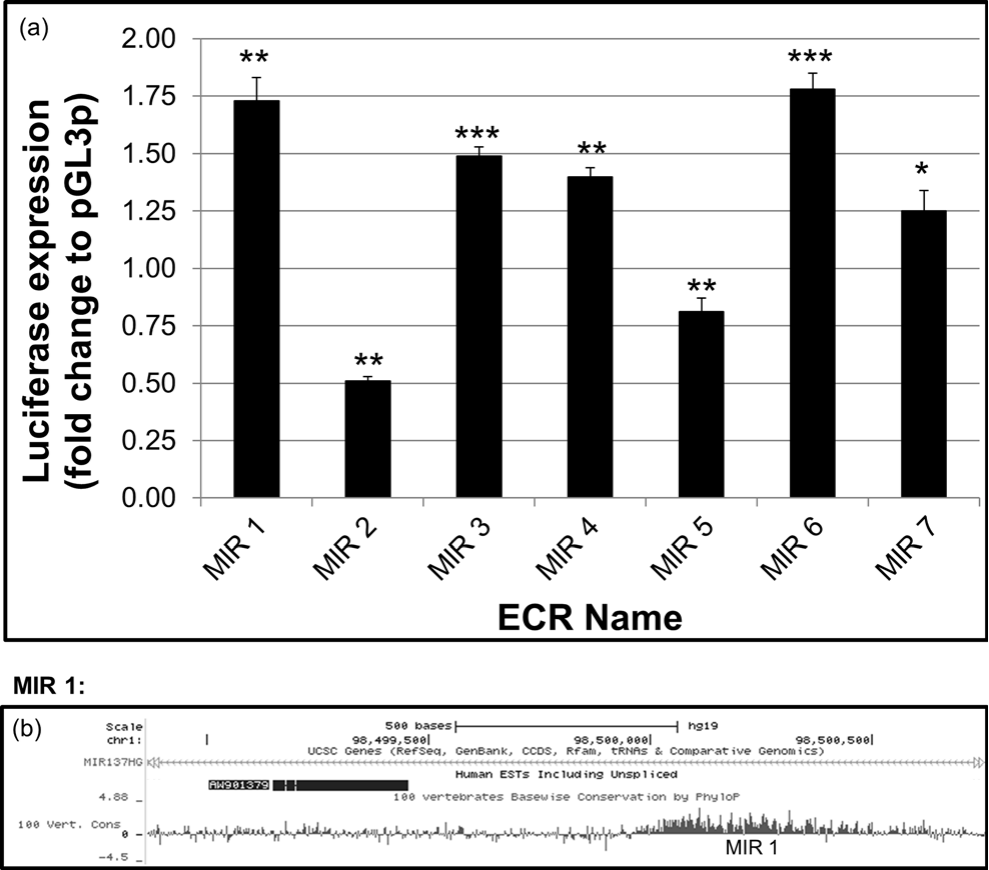
Transcriptional regulatory activity of MIR ECRs: **a**) Regulatory function of ECR sequences was assessed by dual luciferase assay in the pGL3P reporter vector in SH-SY5Y neuroblastoma cells under basal conditions. All ECRs tested displayed regulatory properties in vitro, with 5 showing positive regulatory function and the remaining two displaying negative regulatory effects. *N* = 4; *P < 0.1, **P < 0.01, ***P < 0.001. **b**) Schematic of the MIR 1 ECR and flanking region, showing the human EST, AW901379. AceView (http://www.ncbi.nlm.nih.gov/ieb/research/acembly/) lists this EST as being identified in nervous tissue, and data from Fig. 3a showed histone modifications around the MIR 1 ECR indicative of active transcription from this locus in the human brain

The seven selected ECRs were cloned into pGL3P luciferase reporter vector, and potential transcriptional regulatory function was verified by dual luciferase reporter assay in the SH-SY5Y neuroblastoma cell line (Fig. 4a). When compared to the baseline expression of luciferase from the unmodified pGL3P vector containing a minimal promoter alone, five of the seven selected ECRs were shown to act as positive regulators of reporter gene expression (MIR 1, 3, 4, 6, 7). The two remaining ECRs (MIR 2 and 5) decreased expression of the reporter gene. MIR 1 and MIR 6 were found to be the most active ECRs in our reporter gene assay in the neuroblastoma cell line, SH-SY5Y.

As the MIR137 locus is shown to be highly associated with schizophrenia through GWAS, new ESTs and RNAs from within this region warrant further study for their potential involvement in brain development and function. In this regard, further bioinformatic investigation of the MIR 1 region using GenBank data on human ESTs showed an uncharacterised transcript (AW901379) adjacent to this ECR, identified in nervous tissue (Fig. 4b). Histone modification data across this locus from the Roadmap Epigenomics Consortium showed that MIR 1 displayed H3K4me1 modifications in human embryonic stem cells and derived neuronal progenitor cells, consistent with this site being a poised or active transcriptional regulator in these cells. The same region also displayed H3K4me1 modifications in the foetal brain, as well as seven of the eight brain regions tested, with H3K4me3 marks (indicative of actively transcribed promoter regions) seen in the hippocampus, cingulate gyrus, inferior temporal lobe, angular gyrus, dorsolateral prefrontal cortex and foetal brain (Fig. 3a). This evidence suggests that the MIR 1 ECR may act as a promoter or modulator of expression for an uncharacterised RNA at this locus in CNS tissues.

## Discussion

Analysis of GWAS data has demonstrated that many non-coding regions of the genome are implicated in genetic susceptibility to psychiatric illness (Ripke et al. 2013). This might suggest that many of these associations are highlighting regulatory mechanisms that modulate tissue-specific or stimulus-inducible regulation of gene expression or RNA processing (Quinn et al. 2013). This would be consistent with the episodic nature of many psychiatric conditions, in which regulatory mechanisms would be affected by an environmental challenge, thereby highlighting a major role for gene-environment interactions in such disorders.

In our study, we identified seven non-coding ECRs with potential transcriptional regulatory function at the schizophrenia-associated MIR137 locus (Fig. 1). GWAS SNPs within MIR 3 and 7, and adjacent to MIR 1, 2 and 5, would support a functional role for these elements in the regulation of expression from this locus with relevance to schizophrenia (Fig. 2). Data from the Epigenomics Roadmap Consortium on the chromatin and histone modifications across the ECRs showed that MIR 1, 2 and 6 are predicted to function as active regulatory elements in multiple brain tissues. This supports a role for these ECRs in regulating expression from this locus in the human brain. Further data also suggested and that MIR 3, 4 and 5 are functionally active in both embryonic and induced stem cell lines, as well as stem cell derived neurons (Fig. 3). The data banks we have accessed may therefore point to different times in development and in the adult when our potential regulators would be active. This data may be useful in delineating mechanisms in the development of schizophrenia, e.g. that MIR 3, 4 and 5 may be important in the foetus and therefore focus our model for their function on the developmental aspects of schizophrenia.

We have demonstrated through reporter gene assays that the seven ECRs selected at the MIR137 locus can modulate reporter gene expression in the neuroblastoma cell line, SH-SY5Y (Fig. 4). All but one of the ECRs (MIR 5) is conserved at least to the chicken genome, and we and others have demonstrated that similar evolutionary conservation has identified domains that can support tissue specific marker gene expression in mouse transgenic models (Davidson et al. 2011; Davidson et al. 2006) and in the chicken (Khursheed et al. 2015). In addition, expression data from GenBank suggests an uncharacterised, nervous tissue-expressed transcript originating from the MIR 1 locus, with histone modification data indicating active transcription around this ECR in multiple regions of the human brain (Fig. 3a and 4b).

## Conclusion

In conclusion, bioinformatic analysis identified seven highly conserved, functional ECRs at the MIR137 locus. The transcriptional regulatory activity of the ECRs, predicted from available ENCODE and expression data, was validated by reporter gene assay, and in conjunction with supporting data from the PGC schizophrenia GWAS studies, suggests a functional role for these sequences in the regulation of expression at the MIR137 locus. This demonstrates multiple ‘gene x environment’ pathways that could impact on MIR137 expression levels either individually or synergistically to modulate CNS behaviour, contributing to schizophrenia and potentially other brain-related conditions.
